# Long-Term Maintenance and Meiotic Entry of Early Germ Cells in Murine Testicular Organoids Functionalized by 3D Printed Scaffolds and Air-Medium Interface Cultivation

**DOI:** 10.3389/fphys.2021.757565

**Published:** 2021-12-24

**Authors:** Guillaume Richer, Robin M. Hobbs, Katherine L. Loveland, Ellen Goossens, Yoni Baert

**Affiliations:** ^1^Biology of the Testis Lab, Vrije Universiteit Brussel (VUB), University Medical Campus, Brussels, Belgium; ^2^Centre for Reproductive Health, Hudson Institute of Medical Research, Clayton, VIC, Australia; ^3^Department of Molecular and Translational Sciences, School of Clinical Sciences, Monash Medical Centre, Monash University, Clayton, VIC, Australia

**Keywords:** organoid, 3D printing, testis, spermatogonial stem cells, germline stem cells, tubulogenesis, *in vitro* spermatogenesis

## Abstract

Short-term germ cell survival and central tissue degeneration limit organoid cultures. Here, testicular organoids (TOs) were generated from two different mouse strains in 3D printed one-layer scaffolds (1LS) at the air-medium interface displaying tubule-like structures and Leydig cell functionality supporting long-term survival and differentiation of germ cells to the meiotic phase. Chimeric TOs, consisting of a mixture of primary testicular cells and EGFP^+^ germline stem (GS) cells, were cultured in two-layer scaffolds (2LSs) for better entrapment. They showed an improved spheroidal morphology consisting of one intact tubule-like structure and surrounding interstitium, representing the functional unit of a testis. However, GS cells did not survive long-term culture. Consequently, further optimization of the culture medium is required to enhance the maintenance and differentiation of germ cells. The opportunities TOs offer to manipulate somatic and germ cells are essential for the study of male infertility and the search for potential therapies.

## Introduction

Spermatogenesis is the stepwise process of sperm development within the seminiferous tubules of the testis. The tubules are delimited by a basement membrane and contractile myoid cells. In the tubular compartment, Sertoli cells directly nurture germ cells through the different stages of spermatogenesis: from spermatogonial stem cell (SSC) at the basement membrane to post-meiotic spermatids toward the lumen. The fate of germ cells also relies on secretions coming from the interstitial compartment between the tubules such as testosterone produced by the Leydig cells (Oliver and Stukenborg, 2019). Because of the highly coordinated nature of signaling cues in the testicular microenvironment, it is pivotal for cultures for *in vitro* spermatogenesis (IVS) to reestablish conditions that mimic the two structural compartments of the testis (Oliver and Stukenborg, 2019).

Organ cultures of testicular tissue fragments have been successfully used for IVS in rodents because they best preserve the SSC niche (Sato et al., 2011). However, they do not offer the ability to access and manipulate single cells, making it an inefficient approach for mechanistic studies *ex vivo*. In contrast, testicular organoids (TOs) are customizable and therefore permit detailed analysis of mechanisms underlying testis development and function (Alves-Lopes and Stukenborg, 2018). TOs are 3D multicellular testis surrogates originating from the assembly of cells in suspension. Their fabrication mostly relied on 3D scaffolds made of extracellular matrix (ECM) because of the provision of instructive and structural support to the reorganizing cells (Richer et al., 2020). For instance, tubulogenesis was reported in collagen and Matrigel-based scaffolds, which were not able to support complete spermatogenesis (Zhang et al., 2014; Alves-Lopes et al., 2017). Moreover, the animal and carcinogenic origin of commercially available matrices impedes their clinical application. To overcome these limitations and more closely mimic the native testicular tissue, decellularized testicular matrices were employed (Baert et al., 2017; Pendergraft et al., 2017; Topraggaleh et al., 2019; Vermeulen et al., 2019). Still, although testicular cells from piglets reorganized into TOs with compartmentalized tubule-like structures in porcine-derived testicular matrix, complete spermatogenesis was not observed (Vermeulen et al., 2019). Interestingly, 3D ECM-free approaches also resulted in the formation of compartmentalized organoids from mouse testicular cells, challenging the idea that TO formation depends on external matrices (Yokonishi et al., 2013; Sakib et al., 2019; Edmonds and Woodruff, 2020). Edmonds and Woodruff (2020) elegantly examined the requirement for exogenous ECM-support for TO formation from prepubertal mouse testicular cells in both 2D and 3D immersion culture conditions. In their study, testicular cell self-assembly and self-reorganization were optimal in 3D ECM-free culture and, thus, relied solely on the intrinsic morphogenic capacity of the dissociated immature cells themselves. It was suggested that non-cellular material in the ECM-based culture may have hindered intercellular interactions (Edmonds and Woodruff, 2020). However, germ cells were rarely observed after 14 days in culture (Edmonds and Woodruff, 2020). In contrast, Yokonishi et al. (2013) were able to maintain primary germ cells and germline stem (GS) cells (precultured SSCs) for 61 days and 8 weeks, respectively, in tubule-like structures of millimeter-sized TOs at the air-medium interface (Yokonishi et al., 2013). Noteworthy, cultivation of large tissues imposes the risk of central degeneration, typically occurring in organ culture because of insufficient nutrient supply and anoxia (Kojima et al., 2018). Meanwhile, the use of too low testicular cell densities was shown to result in small spheroidal-shaped aggregates having a reversed architecture (inside-out) or even completely lacking a genuine testicular architecture, likely due to limited intercellular interactions coming from excessive miniaturization (Baert et al., 2019; Sakib et al., 2019).

To restore testicular architecture and avoid degeneration of the organoid core in order to ultimately improve IVS, the current study aimed at forming TOs from prepubertal mouse testicular cells at the air-medium interface in defined 3D printed macropores. The macroporous scaffolds solely served as a delimitation of the area in which the cells can reorganize, with the purpose of scaling the TOs to one functional unit of the testes, representing a seminiferous tubule with surrounding interstitium. Moreover, strain-dependent differences of testicular cells to reorganize and function were examined, as these features affect the efficiency of IVS in organ culture (Portela et al., 2019). Lastly, the possibility to grow GS cells in TOs was investigated, as their use would extend the applicability of TOs in research and development.

## Materials and Methods

### Mice

Testes were obtained from prepubertal wild-type C57BL/6 J or hybrid CBAB6F1 pups (4–5 days old) from the institutional breeding facility (LA2230395: Vrije Universiteit Brussel; SABL063: Monash Animal Research Platform). Following isolation and mechanical removal of the tunica albuginea, testes were cryopreserved by uncontrolled slow freezing as previously described (Baert et al., 2012). Transgenic GS cells were derived from newborn Tg(CAG-EGFP)131Osb males according to an established method (Kanatsu-Shinohara et al., 2003; Kanatsu-Shinohara, 2005). In these mice, the EGFP transgene is driven by the cytomegalovirus intermediate early enhancer coupled to the chicken beta actin promoter, resulting in ubiquitous expression of a green fluorescent reporter. Experimental procedures and animal breeding were approved by the Ethical Committee for the use of Laboratory Animals of the Vrije Universiteit Brussel (permission 19-216-1 under license LA1230216) and Monash Medical Centre B Animal Ethics Committee (MMCB/2019/01, under license SPPL20173).

### Testicular Cell Isolation

Cryopreserved testes were removed from storage after 1–6 months and thawed for 2 min in a water bath at 37°C. Cryoprotectant was diluted by washing the testes twice in Dulbecco’s Modified Eagle’s Medium (DMEM)/F12 containing 10% (v/v) fetal calf serum (10500-056; Thermo Fisher, Merelbeke, Belgium). To obtain single cell suspensions, testes were enzymatically digested using 1.0 mg/ml collagenase Ia (C9891; Sigma-Aldrich, Diegem, Belgium), 0.5 mg/ml hyaluronidase (H3506; Sigma-Aldrich), and 0.5 mg/ml desoxyribonuclease (DN25, Sigma-Aldrich) in Minimum Essential Medium-alpha (αMEM, 32571028; Thermo Fisher; Baert et al., 2019). Cell aggregates were removed by filtration with a 40 μm cell strainer (35234; BD Falcon, Leuven, Belgium). After digestion, cell viability was assessed using the 0.4% trypan blue exclusion test (15250061; Thermo Fisher) in a Neubauer chamber (Neubauer, Blaubrand, Germany) and ranged from 78 to 84%. The final cell seeding densities only took the concentrations of viable cells into account.

### Germline Stem Cell Culture

The GS cell culture was set up according to a previously established protocol (Kanatsu-Shinohara et al., 2003, 2005). Briefly, following enzymatic digestion, testicular cell suspensions from newborn Tg(CAG-EGFP)131Osb males were cultured on 0.2% (w/v) gelatin-coated dishes and passaged to remove most somatic cells which adhered to the coated dishes. The floating cells, enriched for GS cells, were cultured on inactivated mouse embryonic fibroblast feeder cells in GS cell medium (Kanatsu-Shinohara et al., 2003; Kanatsu-Shinohara, 2005). The GS cell cultures were kept at 37°C with 5% CO_2_ in air and passaged weekly, depending on the proliferation rate of GS cells (Kanatsu-Shinohara, 2005). Medium change was performed every 2–3 days.

### Design and Printing of Scaffolds

**Figure 1:**
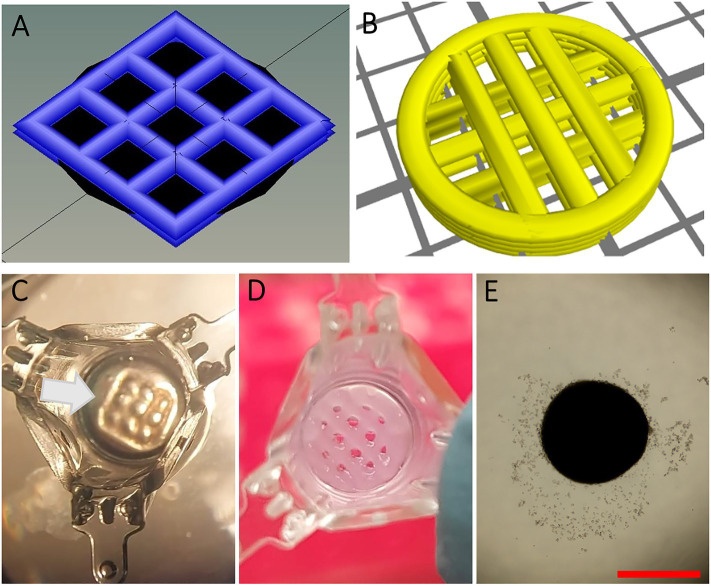
Design and preparation of scaffolds. **(A-B)** 3D design of square-shaped one-layer scaffolds (1LSs, **A**) and circular two-layer scaffolds (2LSs, **B**). **(C)** To ensure proper cell seeding inside its macropores, 1LSs were transferred inside agarose sockets (white arrow) in culture inserts. **(D)** Because of their diameter size, 2LSs were directly transferred in the culture inserts. **(E)** A control condition without 3D printed scaffold was included. Bar = 500 µm.

A square-shaped one-layer scaffold (1LS) with regularly spaced pores in a grid-like pattern (3.5 mm × 3.5 mm, 0.15 mm height with a strut distance of 1.1 mm, strand thickness of 0.6 mm and angle change of 90°) and a mold (4.2 mm × 4.2 mm squares, 1.5 mm height with no spacing between the struts) were designed with Sketchup software (Trimble, Sunnyvale, United States). 3D printing STL files were repaired with Microsoft’s Azure (Microsoft, Redmond, United States) and sliced into a G-code using Heartware software (Cellink, Göteborg, Sweden; Figure 1A). Design and slicing of a circular two-layer scaffold (2LS; 7 mm diameter, 0.6 mm height and grid-like pattern with strut distance of 1.3 mm, strand thickness of 0.6 mm and angle change of 90°) were done with GeSIM Robotics v.1.4.0 software (Figure 1B). Cellink-RGD bioink (nanocellulose-alginate hydrogel with additional arginine-glycine-aspartic acid cell-attachment motifs) was used for printing 1LSs, molds and 2LSs, while regular Cellink bioink lacking RGD binding motifs (Cellink) was also used for 2LSs to promote confinement of the cells within the macropores. The hydrogels were extruded at room temperature from a 25 gauge conical nozzle (Subrex, Carlsbad, United States) onto glass slides using a pneumatic extrusion-based Inkredible+ bioprinter (Cellink) for 1LSs and the molds or the pneumatic extrusion-based Bioscaffolder (SYSENG, Salzgitter, Germany) for 2LSs. Extruding pressures of the printing process ranged from 5 to 15 kPa and 24 kPa, and the printing speed from 17.5 to 20 mm s^−1^ and 13 to 20 mm s^−1^, respectively. To allow polymerization of the bioink, the printed hydrogels were crosslinked for 5 min with 100 mM CaCl_2_ (Cellink), followed by a rinse with αMEM.

### Scaffold Preparation

To ensure proper cell seeding inside the printed macropores, 1LSs (3.5 mm^2^) were placed inside agarose sockets (Baert et al., 2019). For this, a 0.35% (w/v) gel stand was created by placing 100 μl of molten agarose (16500100; Thermo Fisher) in 24-well plate hanging culture inserts with a diameter of 7 mm (MCRP24H48; Sigma-Aldrich). When agarose had gelled, crosslinked molds (4.2 mm^2^) were placed onto the gel stands. Then, 25 μl of molten agarose was pipetted around the molds and left to harden. The removal of the molds resulted in agarose sockets (Figure 1C, white arrow) in which crosslinked 1LSs were positioned using a spatula. Because of their size (7 mm diameter), 2LSs were directly transferred onto agarose gel stands placed in the culture inserts (Figure 1D).

### Testicular Organoid Culture

TOs were prepared by seeding 1 × 10^6^ primary testicular cells (±three pups) in a 30 μl drop of basal medium onto 1LSs and control agarose stands without scaffold (Figure 1E) (C57BL/6 J, *n* = 5 technical replicates; CBAB6F1, *n* = 3;) on one hand, or a chimeric mixture of testicular cells from prepubertal C57BL/6 J mice and cultured GS cells onto 2LSs at a ratio of 2:1 (*n* = 3 technical replicates per condition) on the other (Yokonishi et al., 2013). Primary and chimeric TOs were cultured for up to 6 weeks at the air-medium interface at 35°C in a humidified atmosphere with a normal O_2_ tension (21%) and 5% CO_2_. The basolateral compartment of the culture wells was filled with 700 μl basal culture medium consisting of αMEM supplemented with 10% (v/v) KnockOut Serum Replacement (KSR, 10828010; Thermo Fisher), 1% (v/v) Penicillin Streptomycin (1540-122; Thermo Fisher) and 10^−7^ M melatonin (M5250; Sigma-Aldrich), successfully used previously to induce complete spermatogenesis in murine organ and cell culture (Reda et al., 2017; Baert et al., 2019). Additionally, for chimeric TOs during long-term culture, basal medium was supplemented with 10^−6^ M retinol (R7632; Sigma-Aldrich) as precursor of retinoic acid, which has been shown to improve spermatogenesis from SSCs and cultured pluripotent stem cells (Arkoun et al., 2015; Wang et al., 2016). Retinol was added every second day and medium was refreshed and collected weekly for hormone analyses. Gross morphology was evaluated using a stereomicroscope. The graphics depicting the primary TO and chimeric TO culture set ups were created with BioRender.com (RY23APYYXPS).

### Immunocytochemistry

Prior to initiation of the TO cultures, testicular cells obtained after the enzymatic digestion of testes from C57BL/6 J or CBAB6F1 mice were re-suspended at 1 × 10^6^ cell/ml of cytospin buffer containing phosphate-buffered saline [PBS (1X), 70011051; ThermoFisher] and 1% (w/v) bovine serum albumin (10735094001; Roche). Around 30 μl of the cell suspension was loaded in cytofunnels and centrifuged onto glass slides (J1800AMNZ; VWR) at 1350 rpm for 3 min. Subsequently, the slides were dried at room temperature, fixed for 10 min in 4% formaldehyde at room temperature and subjected to heat-mediated epitope retrieval at 95°C in 10 mM Tris-EDTA buffer (pH 9.0). Following three washes of 5 min in PBS, the slides were blocked with 5% serum and then incubated overnight in a humid chamber at 4°C with mouse monoclonal anti-phospho-histone H2A.X (γH2AX, 05-636-I; Sigma Aldrich, 1:100) and rabbit polyclonal anti-DEAD-box helicase 4 (DDX4, ab13840; Abcam, 1:400) primary antibodies diluted in PBS. The following day, the slides were washed with PBS and incubated for 1 h at room temperature with donkey anti-rabbit 488 (A21206; Life technologies, 1:200) and donkey anti-mouse 594 (A21203; Life Technologies) secondaries in PBS. Following additional PBS washes, the slides were mounted with Prolong Gold Antifade Mountant with DAPI (P36931; Thermo Fisher) and stored at 4°C until evaluation.

### Histological and Immunofluorescent Analyses

Following fixation in acidified alcoholic formalin (PFAFA0060AF59001; Labonord), primary TOs in 1LSs were captured in 2% (v/v) agarose, dehydrated to 70% ethanol and embedded in paraffin. Paraffin blocks were sliced into 5 μm thick serial sections (SM2010R; Leica). The slides were deparaffinized in xylene and rehydrated in descending concentrations of isopropanol (100, 100, 90, and 70%). Following a 5 min wash in PBS, the sections were stained with periodic acid-Schiff (PAS) and hematoxylin to perform gross morphological evaluations of the samples. Chimeric TOs were handled differently: after 1 or 6 weeks of culture, they were fixed with 4% formaldehyde in PBS and cryo-embedded in optimal temperature cutting compound to preserve the EGFP signal. Eight μm thick serial frozen sections were stained with Masson’s trichome for representative images of the samples. For in-depth analysis of primary and chimeric TOs, immunofluorescent staining was performed to detect key phenotypical and functional testicular markers: laminin (LN, marks basement membrane), zona occludens 1 (ZO1, tight junctions), 3beta-hydroxysteroid dehydrogenase (3ß-HSD, Leydig cells), actin alpha 2 (ACTA2, myoid cells), SRY-Box transcription factor 9 (SOX9, Sertoli cells), DDX4 (germ cells), γH2AX (meiotic spermatocytes), cAMP-responsive element modulator (CREM, spermatids), and peanut agglutinin (PNA, acrosome). Detailed protocols are listed in Table 1. Briefly, slides were subjected to epitope retrieval at 95°C in 10 mM Tris-EDTA buffer (pH 9.0), followed by three washes of 5 min in PBS and then blocked with 5% serum in PBS at room temperature. The slides were then incubated overnight in a humid chamber with primary antibodies diluted in PBS at 4°C, and the following day, washed with PBS and incubated with the secondary antibodies diluted in PBS. Following additional PBS washes, the slides were mounted with Prolong Gold Antifade Mountant with DAPI or SYTOX Deep Red Nucleic Acid Stain (P36987; Thermo Fisher) and stored at 4°C until evaluation. For the identification of apoptotic cells, a terminal deoxynucleotidyl transferase-mediated dUTP nick end labeling (TUNEL, *In Situ* Cell Death Detection Kit, Fluorescein, 11684795910; Roche) assay was performed according to the manufacturer instructions with an enzyme solution diluted 1:10 in the TUNEL reaction mix. Testis sections from adult mice were used as positive controls for the testicular markers, while sections treated with 1,500 U/ml DNase I recombinant were used as positive controls for the TUNEL assay. As negative controls, samples were incubated with PBS or the TUNEL reaction mix only, omitting the primary antibodies or the terminal deoxynucleotidyl transferase enzyme solution, respectively. Representative bright field and fluorescence images were taken on an inverted Olympus or Leica microscope. ImageJ software (National Institute of Health) was used to stack and analyze the images. In addition, germ cell differentiation inside tubule-like structures of primary TOs was analyzed with an Axio Scan Z.1 slide scanner (Carl Zeiss) and Zen Lite software (Carl Zeiss).

**Table 1:** Antibody information and reagents used for immunofluorescent stainings.

Protein	Target	Washing buffer (3 × 5 min at room temperature)	Epitope retrieval 75 (paraffin-embedded) or 30 (frozen) min at 95°C]	Blocking (1 h at room temperature)	Primary antibody (overnight at 4°C)	Primary antibody type	Primary antibody dilution	Secondary antibody (1 h at 4°C)
SOX9	Sertoli cells	0.05% Tween-20 in PBS	TE buffer^a^	5% NDS^b^	AB5535^c^	Rabbit pAb	1:200	Donkey anti rabbit 488^g^
ACTA2	Myoid cells	PBS	TE buffer	5% NDS	A2547^d^	Mouse mAb	1:400	Donkey anti mouse 594^g^
yH2AX	Meiotic cells	PBS	TE buffer	5% NDS	05-636-I^d^	Mouse mAb	1:100	Donkey anti rabbit 594^g^
3β-HSD	Leydig cells	PBS	TE buffer	5% NDS	15516-1-A^e^	Rabbit pAb	1:50	Donkey anti rabbit 488^g^
DDX4	Germ cells	PBS	TE buffer	5% NDS	AF2030^f^	Goat pAb	1:100	Donkey anti goat 647^g^
DDX4	Germ cells	PBS	TE buffer	5% NDS	ab13840^g^	Rabbit pAb	1:400	Donkey anti rabbit 488
ZO1	Tight junctions	PBS	TE buffer	5% NDS	617300^h^	Rabbit pAb	1:200	Donkey anti rabbit 488
LN	Laminin	PBS	TE buffer	5% NDS	ab11575^g^	Rabbit pAb	1:100	Donkey anti rabbit 488
CREM	Post-meiotic cells	PBS	TE buffer	5% NDS	sc-440^i^	Rabbit pAb	1:400	Donkey anti rabbit 488

a 10 mM Tris base, 1 mM EDTA solution, 0.05% Tween-20, and pH 9.0.

b Jackson ImmunoResearch Europe Ltd., Sufford, United Kingdom.

c Millipore, Overijse, Belgium.

d Sigma-Aldrich, Diegem, Belgium.

e Proteintech, Manchester, United Kingdom.

f R&D systems, Abingdon, United Kingdom.

g Abcam, Cambridge, United Kingdom.

h Thermo Fisher, Merelbeke, Belgium.

i Santa Cruz, Heidelberg, Germany.

### Functionality of Leydig Cells

Concentrations of testosterone were assessed with the Elecsys Testosterone II competitive immunoassay in a Cobas 8000 bioanalyzer (Roche Diagnostics). Briefly, 20 μl of the sample was incubated with a biotinylated monoclonal testosterone-specific antibody. Streptavidin-coated microparticles and a testosterone derivative labeled with a ruthenium complex were added in order to bind the formed complex to the solid phase *via* interaction of biotin and streptavidin. The reaction mixture was aspirated into the measuring wells, where the microparticles were magnetically captured onto the surface of the electrode. Unbound substances were then removed with ProCell/ProCell M (Roche Diagnostics). Hormone concentrations were assessed by electrochemiluminescence, measured by a photomultiplier with a functional sensitivity of the assay of 0.120 ng/ml.

### Quantification of Germ Cell Types

Quantification of γH2AX and DDX4^+^ germ cells was performed prior to culture (day 0) and week 6 of culture on cytospins and TO sections, respectively. Pre-meiotic (DDX4^+^/γH2AX^−^) and meiotic (DDX4^+^/γH2AX^+^) leptonema and zygonema were identified based on the cytoplasmic expression of DDX4 and the nuclear distribution of γH2AX. Three representative fields per cytospin slide (CBAB66F1 and C57BL/6 J: *n* = 3) were imaged at 20X on an inverted Olympus microscope. ImageJ software (National Institute of Health) was used to stack and count the amount of positive and negative cells on each image. For the TOs, independent cross-sections (minimum 25 μm apart) of every technical replicate (CBAB66F1: *n* = 3; C57BL/6 J: *n* = 5) were analyzed with Zen Lite software (Carl Zeiss). The percentages of calculated germ cells per strain were plotted in graphs for each time point. Additionally, the results were expressed as the ratio between the number of DDX4^+^ germ cells in the newly formed tubule-like structures and the total tubule area (mm^2^) of the organoids, per strain. Finally, the relative percentages of germ cell populations (pre-meiotic, leptonema, zygonema) in the TOs were calculated.

### Statistical Analyses

Statistical analyses were performed using Prism software (GraphPad Prism 8). The influence of culture period and mouse strain on germ cell numbers were determined by mixed-effects analysis followed by Sidak’s multiple comparisons test. The influence of mouse strain on germ cell numbers/tubular area was determined by Mann-Whitney test. Concentrations of testosterone over time and between the mouse strains at weekly timepoints were analyzed using a repeated measures two-way ANOVA followed by a Tukey’s *post hoc* test. Results are presented as single dots, mean and SD. Statistical significance was set at *p* < 0.05.

## Results

### Primary TOs From Two Different Mouse Strains Display Similar Characteristics During Long-Term Culture in Cellink-RGD 1LS

#### 1LS-TOs Exhibit a Compartmentalized Testicular Architecture With Re-establishment of the SSC Niche Components, but Also a Heterogeneous Morphology and Inconsistent Tissue Health

**Figure 2:**
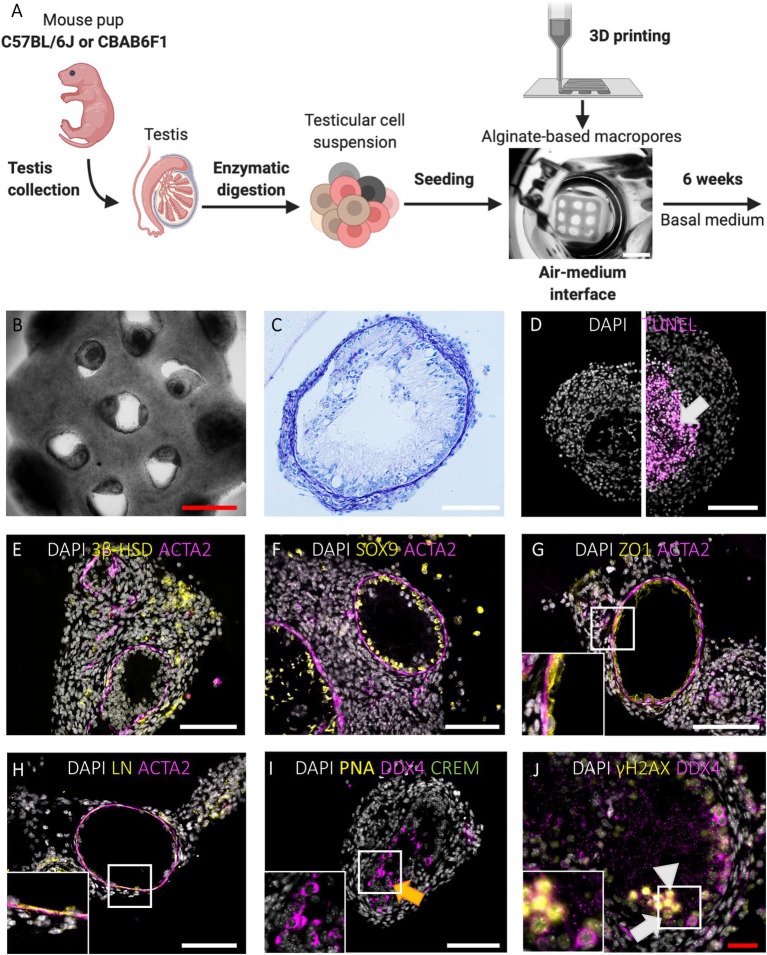
Primary TOs in 1LS of both mouse strains restore in vivo testicular histology and support long-term survival and meiotic entry of early germ cells but display heterogeneous morphology. **(A)** Schematic representation of experimental procedure. Bar = 2 mm. **(B-C)** Gross morphological analysis of constructed TOs by bright field appearance **(B)** and PAS/Hematoxylin staining **(C)**. Bars = 500 µm (red) and 100 µm (white). **(D)** TUNEL assay revealed cell death in the central tissue region when early aggregates overgrew the macropores of 1LS to fuse into larger TOs (arrow). Bar = 100 µm. **(E-H)** Immunofluorescent staining of SSC niche cells and extracellular matrix: Leydig cells (3ß-HSD, **E**), Sertoli cells (SOX9, **F**), blood-testis-barrier (ZO1, **G**), laminin (LN, **H**), and peritubular myoid cells (ACTA2, **E-H**) are shown. Inserts correspond to boxed areas **(G-H)**. Bars = 100 µm. **(I,J)** Immunofluorescent staining of TOs for the constitutive germ cell marker DDX4 **(I-J)**, the post-meiotic germ cell markers CREM and PNA **(I)** and the meiotic marker γH2AX **(J)**. Orange arrow represents presumptive spermatocytes **(I)**. Boxed area includes leptotene (white triangle) and zygotene spermatocytes (white arrow) **(J)**. Bars = 100 µm (white) and 50 µm (red).

When primary testicular cells were cultured on agarose stands without delimitation, they self-reorganized into structures without size restrictions (Figure 1E). To delimitate the area in which testicular cells reorganize, macroporous (pore diameter of 473,8 ± 99,2 μm) 1LSs were printed composed of cell-interactive Cellink-RGD (Figures 1A,C, 2A; Baert et al., 2019). After 6 weeks in basal medium, testicular cells from both mouse strains had self-reorganized into compartmentalized organoids with one or more distinct tubule-like structures with lumen, epithelium, and tubular wall, surrounded by an interstitium (Figures 2B,C). These TOs were healthy in the sense that there were no signs of focalized cell death (Figure 2D, left panel). However, when cells from adjacent pores aggregated, large TOs displaying signs of core degeneration were formed (Figure 2D, right panel; Supplementary Figure 1A). Tubule-like structures of TOs in 1LSs from both strains displayed similar somatic marker spatial arrangements, resembling their *in vivo* expression profiles (Supplementary Figures 1B–E): Leydig (3ß-HSD^+^) and elongated peritubular myoid (ACTA2^+^) cells reorganized around the basement membrane (LN^+^) of tubules containing Sertoli cells (SOX9^+^) that were interconnected by tight junctions (ZO1^+^; Figures 2E–H).

#### 1LS-TOs Enable Long-Term Survival and Meiotic Entry of Early Germ Cells

**Figure 3:**
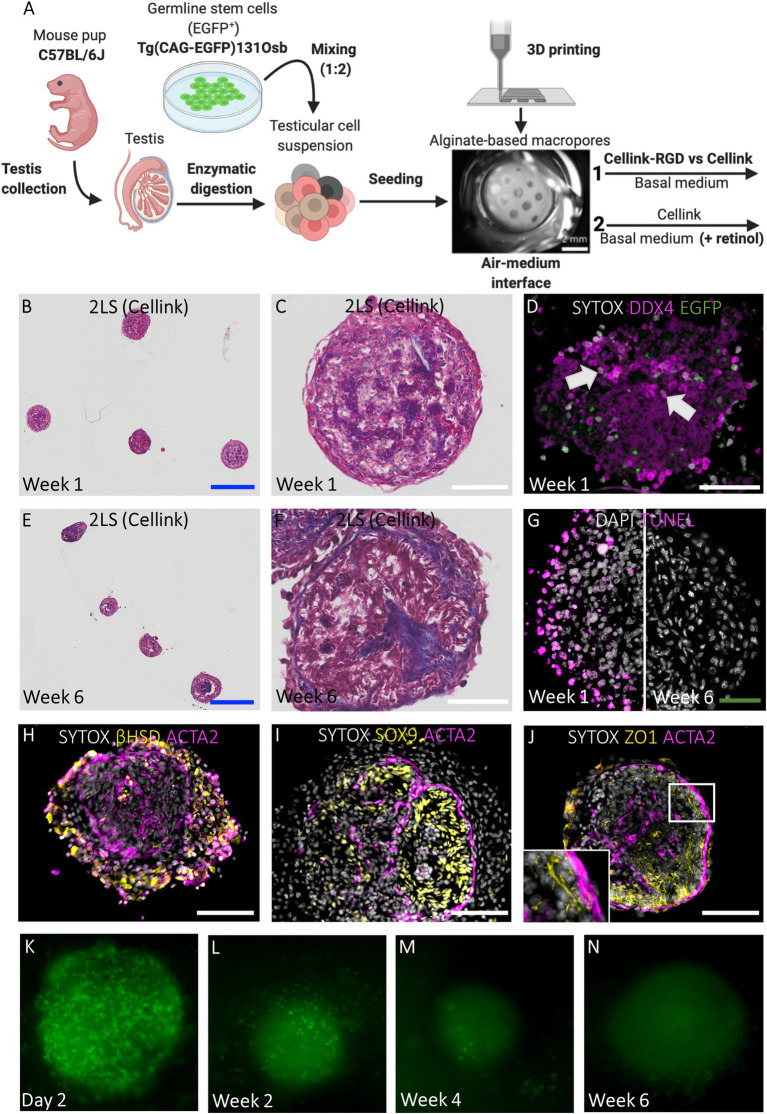
Changing germ cell dynamics and detection of steroidogenic activity in primary 1LS-TOs. **(A)** Effect of time-course and mouse strain (C57BL/6 J: *n* = 5, CBAB6F1: *n* = 3) on relative percentages of germ cell numbers. Asterisks show statistical significance. **(B)** Absolute germ cell numbers per mm^2^ in primary TOs in 1LS at week 6. **(C)** Relative proportion of pre-meiotic and meiotic leptonema and zygonema in primary TOs in 1LS at day 0 and week 6 of culture. **(D)** Comparison of testosterone secretion in primary TOs between mouse strains at each timepoint. Different superscripts (a–f) show statistical significance over time within one mouse strain. Asterisk shows statistical significance between mouse strains on a specific time point.

Germ cell differentiation in the tubule-like structures of primary TOs was assessed by triple immunofluorescence staining for DDX4, CREM, and PNA at week 6. *In vivo*, DDX4 is localized in the cytoplasm of germ cells, with the strongest signal at the spermatocyte stage (Supplementary Figure 1F, red arrowhead). While CREM protein is localized in nuclei, PNA binds to components of the acrosomal matrix, specifically in the cytoplasm of round (DDX4^+^/CREM^+^/PNA^+^: Supplementary Figure 1F, red arrow) and elongated (DDX4^−^/CREM^+^/PNA^+^: Supplementary Figure 1F, red triangle, inset) spermatids. Although no post-meiotic germ cells could be observed as indicated by the absence of CREM and PNA expression, 26 ± 33 (C56BL/6J) and 20 ± 14 (CBAB6F1) DDX4^+^ germ cells per mm^2^ tubular area could still be observed at the basement membrane but also at the apical side of the epithelium, suggesting the presence of meiotic spermatocytes in both strains (Figure 2I, orange arrow; Figures 3A,B). Therefore, the expression of DDX4 and nuclear distribution of γH2AX was used to define the meiotic stage to which spermatocytes had progressed. *In vivo*, leptotene spermatocytes are represented by faint DDX4 and bright γH2AX signals (Supplementary Figure 1G, white triangle, panel 1), while the γH2AX intensity reduces as spermatocytes progress through the zygotene phase (Supplementary Figure 1G, white arrow, panel 2). Pachytene spermatocytes are characterized by a bright DDX4 staining and foci of γH2AX accumulation in the sex vesicles (Supplementary Figure 1G, white arrowhead, panel 3). While all germ cells were pre-meiotic prior to culture, at week 6, more than 50% of the germ cells had progressed to leptotene (Figure 2J, white triangle) or zygotene (Figure 2J, white arrow) spermatocytes (Figures 3B,C). However, pachytene spermatocytes were absent. These findings suggest that meiotic progression was arrested at the zygotene phase in TOs in both strains. Importantly, the fraction of germ cells was significantly lower at week 6 in comparison to the initiation of the culture (C57BL/6 J: *p* = 0.0058; CBAB6F1: *p* = 0.0494) (Figure 3A).

#### 1LS-TOs Support Steroidogenic Activity

The steroidogenic activity of Leydig cells in the TOs was quantified by measuring testosterone concentrations in the culture media throughout the culture period, and fluctuations over time were compared between the strains (Figure 3D). In C57BL/6 J TOs, the testosterone concentration decreased significantly in week 2 (*p* = 0.0042) and 3 (*p* = 0.0220) compared to week 1, but recovered to initial values from the fourth week onwards. In CBAB6F1 TOs, the testosterone concentration reduced significantly at week 2 (*p* = 0.0138) and remained low until the end of the culture period (week 6). Aside from week 1 when C57BL/6 J TOs produced significantly more testosterone (*p* = 0.0071), there was no significant difference in testosterone levels between the strains at other time points.

### Chimeric TOs Can Be Formed in Both Cellink-RGD and Cellink 2LSs

#### Short-Term 2LS Cultured Chimeric TOs Display a Homogeneous Morphology

**Figure 4:**
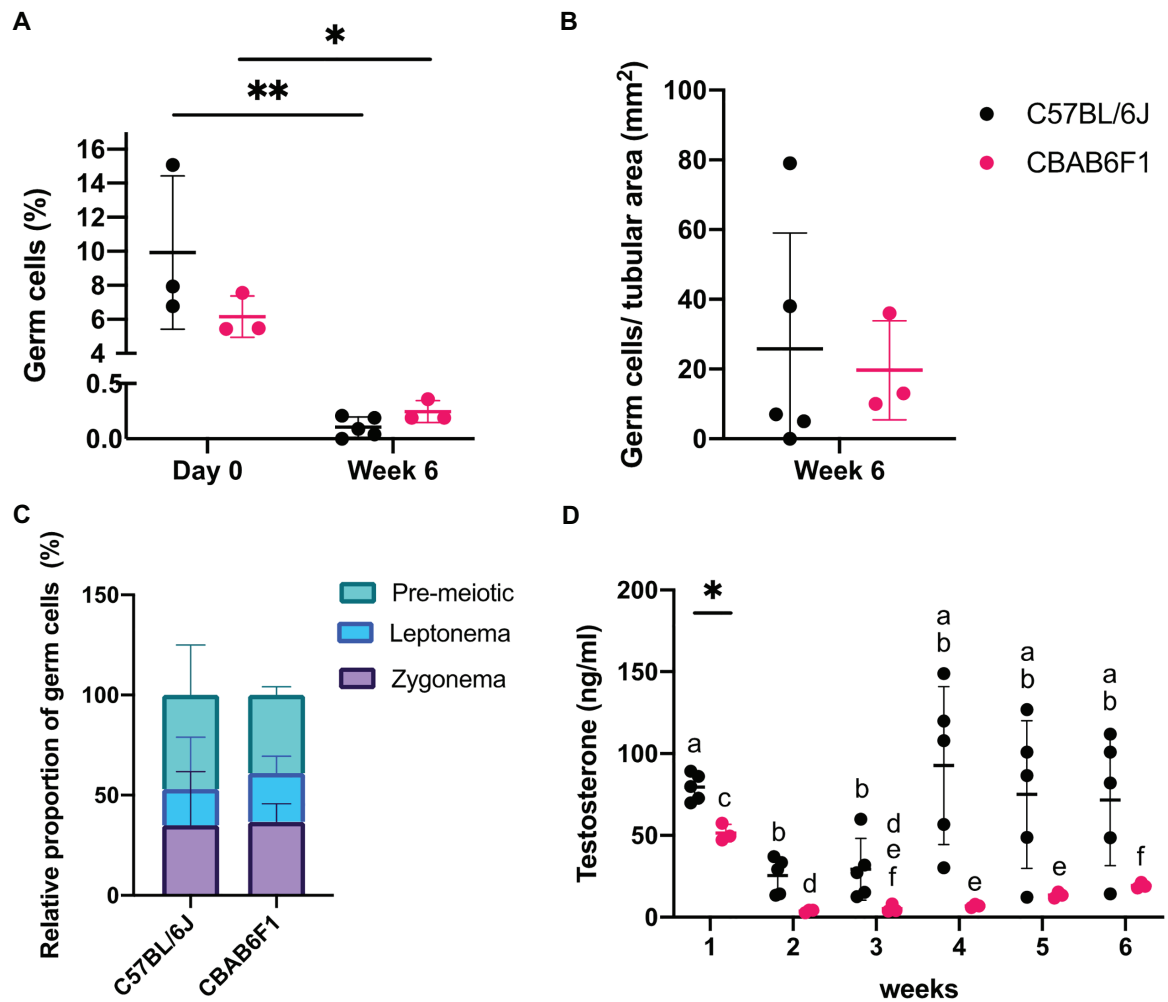
Chimeric TOs in regular 2LSs display uniform morphology and restore components of the SSC niche but show germ cell loss. **(A)** Schematic representation of experimental procedure. First, modified Cellink-RGD and regular Cellink were compared for their ability to immobilize reorganizing chimeric cell mixtures inside the macropores of 2LS (1). Afterwards, basal medium was supplemented with retinol to promote spermatogenesis during long-term culture of TOs in 2LS composed of regular Cellink (2). Bar = 2 mm. **(B, C, E, F)** Histological examination by Masson’s trichrome staining of chimeric TOs in Cellink 2LS after short-term **(B, C)** and long-term **(E, F)** culture. Bars = 500 µm (blue) and 100 µm (white). **(D)** Integration of DDX4+/EGFP+ cells (white arrows) into chimeric TOs after short-term culture. Bar = 100 µm. **(G)** Immunofluorescent TUNEL assay to assess cell death: death cells located at the periphery of 2LS-TOs the first week, but were absent at week 6. Bar = 50 µm. **(H-J)** Immunofluorescent stainings of SSC niche: Leydig cells (3ß-HSD, **H**), Sertoli cells (SOX9, **I**), blood-testis-barrier (ZO1, **J**) and peritubular myoid cells (ACTA2, **H-J**) are shown. Inset corresponds to boxed area **(J)**. Bar = 100 µm. **(K-N)** Non-invasive analysis of EGFP cells during long-term culture: day 2 **(K)**, week 2 **(L)**, week 4 (M), week 6 **(N)**.

The potential to cultivate GS cells in TOs made up of primary testicular cells was tested. For this, testicular cells derived from prepubertal C57BL/6 J mice were mixed at a ratio of 2:1 with GS cells expressing an ubiquitous EGFP reporter transgene (Figure 4A; Supplementary Figures 2A–D). Because cellular aggregates fused to form larger structures exhibiting core degeneration in 1LSs (Figure 2D), the focus was first placed on improving TO morphology using basal medium by adapting the scaffold design. This was evaluated during short-term culture of 1 week [Figures 4A(1)–D]. To immobilize the chimeric cell mixtures inside the macropores during self-reorganization, scaffolds consisting of either Cellink-RGD or regular Cellink and an additional layer of bioink were 3D printed [pore diameter of 531,25 ± 46,22 μm; Figures 1B,D, 4A(1)]. Both regular Cellink (Figures 4B–D) and Cellink-RGD 2LSs (Supplementary Figure 2E) resulted in similar single spheroidal-shaped aggregates (454 ± 100,78 μm in diameter) after 1 week of culture. However, while the aggregates floated freely in Cellink 2LSs (Figures 4B,C), they adhered to Cellink-RGD 2LSs (Supplementary Figure 2E). Nevertheless, the type of hydrogel did not have noticeable effects on the histology, as all samples showed formation of cord-like structures (Figures 4B,C; Supplementary Figure 2E). In addition, EGFP-GS cells positive for DDX4 could be observed in the center and periphery of the chimeric aggregates (Figure 4D, white arrows). Noteworthy, dying cells were detected, but only in the periphery of the developing organoids (Figure 4G).

#### Long-Term 2LS Cultured Chimeric TOs Have a Compartmentalized Testicular Architecture and Consistent Tissue Health With Temporary Integration of GS Cells

Because the type of hydrogel did not affect histology during short-term culture, in the next experiment, the chimeric cell mixtures were cultured for 6 weeks in 2LSs composed of regular Cellink in basal medium with or without retinol supplementation in an attempt to overcome the meiotic block observed in the primary TOs in 1LSs [Figure 4A(2)]. Unlike primary TOs in 1LSs, early aggregates in 2LSs did not outgrow the pores to form larger TOs, but instead developed into uniform spheroidal-shaped TOs measuring 416,36 ± 35,57 μm in diameter without core degeneration at week 6 (Figures 4E–G). Specifically, the chimeric TOs comprised two distinct compartments consisting of interstitial Leydig and peritubular myoid cells or tubular Sertoli cells (Figures 4H–J), separated by a tubular wall (Figures 4E –F). Nonetheless, germ cell differentiation was not observed, regardless of the culture medium; notably, DDX4 and EGFP^+^ cells were completely absent at the end of the culture (Supplementary Figure 2F). Non-invasive follow-up of GS cells showed a dramatic decrease of EGFP+ cells over time (Figures 4K–N). A complete GS loss manifested in week 4 (Figure 4M).

## Discussion

In this study, 3D printed macropores served as a delimitation of the area in which testicular cells can grow in order to optimize TO morphology and histology. The resulting TOs had compartmentalized testicular cells that reorganized to a seminiferous tubule-like structure with surrounding interstitium, representing the functional unit of the testes. These functionalized TOs supported survival of primary germ cells, but not GS cells, for at least 6 weeks along with germ cell differentiation up to the level of early meiosis. Long-term germ cell survival in compartmentalized TOs is already a big step toward achieving complete IVS.

These findings extend our previous work in which lower densities of prepubertal testicular cells from C57BL/6N^ACR-EGFP^ mice formed spheroidal-shaped aggregates in the macropores of 3D printed Cellink-RGD scaffolds without recognizable testicular architecture (Baert et al., 2019). Here, we showed that increased testicular cell densities promoted tubulogenesis, resulting in structurally compartmentalized organoids. The fact that low cell densities were also found to affect organoid morphogenesis in pig, macaque, and human suggests that optimizing this parameter ensures proper TO formation (Sakib et al., 2019). In terms of histology, our 3D printed culture system revised existing TO cultures relying on self-assembly in ECM-free conditions. Complementary to Edmonds and Woodruff (2020) (394 μm in diameter), we also successfully generated small compartmentalized TOs containing only one tubule. Additionally, we extended the germ cell survival to 6 weeks, likely due to cultivation at the air-medium interface. Simultaneous access to air and nutrients has been shown to be critical in organ cultures for germ cell functionality (Sato et al., 2011). Building on their organ culture expertise, Ogawa and colleagues generated millimeter-sized TOs on agarose pillars with, similar to our observations, meiotic cell formation during long-term cultures (Yokonishi et al., 2013). However, the large organoid size may have limited the diffusion of survival factors, resulting in signs of degeneration in the central part of their TOs. It was previously suggested that the integrity of testicular tissue fragments is optimal when they have a diameter of 300–400 μm for proper exchange of O_2_ and nutrients (Komeya et al., 2016). Yet, in the current study, pore outgrowth in 1LSs and fusion of adjacent aggregates resulted in large TOs with uneven morphology and structure, displaying central degeneration and possibly variable testosterone production as well. The switch to 2LS and non-cell-interactive Cellink bioink made of inert alginate-nanocellulose further promoted cell entrapment and, thus, intercellular interactions. The resulting chimeric TOs measured approximatively 416 μm in diameter and did not display central degeneration. Considering uniform TOs hold more consistent readouts, their formation by means of adaptable 3D printed scaffolds would greatly help in developing a robust culture system for basic science and applications in reproductive toxicity testing, drug discovery and fertility preservation.

We generated TOs using cells from the C57BL/6 J and the CBAB6F1 strains, both being popular models for *in vitro* gametogenesis research (Portela et al., 2019; Akin et al., 2021). Although tubule-like structures carrying germ cells were formed in TOs after 6 weeks of culture, germ cells from both strains did not progress through meiosis. This was unexpected since the same culture medium resulted in spermatogenesis in murine organ culture (Reda et al., 2017) and in cellular aggregates from transgenic C57BL/6N^ACR-GFP^ mice in our previous 3D printing model (Baert et al., 2019). Of note, the location of the transgene in the genome of C57BL/6N^ACR-GFP^ mice is unknown (Hasuwa et al., 2010). Also, Substrain-dependent differences exist (Jankovicova et al., 2016). Their influence on IVS has never been studied before and, consequently, cannot be ruled out. Moreover, TOs, which are reassembled testicular cell suspensions, may require an adapted version of the medium used in the organ culture system.

For instance, to determine whether the lack of complete spermatogenesis in our TOs could also in part be attributed to a failure to accurately represent a physiologically relevant testis surrogate, somatic support and Leydig cell function were assessed. Here, we demonstrated the development of a tubular lumen, as well as junctional specializations by Sertoli cells, both of which are signs of post-pubertal maturity. Concerning Leydig cell functionality, testosterone from C57BL/6 J and CBAB6F1 primary TOs showed an unexpected drop after 1 week of culture, which corresponds with the start of puberty in mice at 10 days post-partum (Michael et al., 1980). In C57BL/6 male mice, testosterone was shown to rise from puberty and peaks at 40 days (Bell, 2018). Similar peaks of testosterone were absent in the TO cultures, regardless of the strain. The discrepancy in testosterone secretion patterns between the TOs and *in vivo* levels could (in part) explain the observed meiotic block. Even though it is not necessary in organ cultures to achieve IVS (Sato et al., 2011), in future TO experiments, the steroidogenic activity of Leydig cells could be stimulated by gonadotrophins from day 12 onwards to obtain strong testosterone levels as previously shown (Edmonds and Woodruff, 2020).

Additionally, while tissue handling has been reported to disturb spermatogenesis in organ culture *via* activation of the innate immunity (Abe et al., 2020), the successive processes of freezing-thawing and enzymatic digestion of the mouse testes to form TOs possibly afflicted even greater insult and may need to be countered.

Finally, even though TOs showed somatic cell reorganization, the relatively low abundance of germ cells reveals an inherent disturbance and requires particular attention as spermatogonia are essential determinants of sperm output. In contrast to testicular organ culture in which the architecture is preserved, the absence of a niche for the SSCs during the first days of TO culture may have triggered germ cell apoptosis. Previously, germ cells left without somatic support in aggregates have been reported to be vulnerable *in vitro* (Reda et al., 2014). This further indicates that the culture media will have to be optimized to enhance germ cell maintenance during the first weeks of cell reorganization. In addition to assessing the ability of TOs to support primary germ cells, we also investigated the possibility to grow and differentiate transgenic EGFP^+^ GS cells in chimeric TOs. For this, retinol supplementation was tested as vitamin A metabolites play an essential role in SSC and GS cell differentiation (Arkoun et al., 2015; Wang et al., 2016). Strikingly, the amount of EGFP^+^ germ cells decreased drastically during the first weeks to finally disappear at week 4, hindering the evaluation of retinol supplementation on GS cell differentiation. Compared to our study, EGFP signals lasted longer in the reconstructed tubule-like structures of Ogawa’s group, reaching maxima in weeks 4–8 (Yokonishi et al., 2013). The addition of glial cell line-derived neurotrophic factor during reassembly may have benefited germ cell maintenance because of its known positive effects on SSC self-renewal (Kanatsu-Shinohara et al., 2003). Also, the possibility exists that unfavorable cell ratios could have resulted in inadequate support of germ cells from the somatic cells following the incorporation of GS cells.

To conclude, we reported the formation of TOs with compartmentalized architecture, supporting long-term survival and meiotic entry of early germ cells. Albeit this progress, further optimization of the culture medium will be necessary before full spermatogenesis can be obtained from primary germ cells and GS cells in the described TOs. The opportunity to manipulate multiple cell types in TO cultures will accelerate our understanding of testis development and function, but also enable generation of testicular disease and cancer models (Alves-Lopes and Stukenborg, 2018). In that regard, GS cells can be cultured over several years and are in combinations with TOs a valuable tool for studies on gene functions in spermatogenesis (Kanatsu-Shinohara, 2005; Takehashi et al., 2007). Taken further, germline genome editing in TO cultures may one day permit patients suffering from a genetic condition to father healthy children with healthy sperm. Finally, the use of TOs as a drug screening model will reduce the number of animals for the high demanding reproductive toxicity studies (Rovida, 2009).

## Data Availability Statement

The original contributions presented in the study are included in the article/Supplementary Material, further inquiries can be directed to the corresponding author.

## Ethics Statement

The animal study was reviewed and approved by Ethical Committee for the use of Laboratory Animals of the Vrije Universiteit Brussel Monash Medical Centre B Animal Ethics Committee.

## Author Contributions

GR, RH, KL, EG, and YB designed the study. GR and YB performed the experiments, the analyses, and interpretation of the data. YB and EG obtained funding. GR wrote the manuscript. YB supervised the project. All authors contributed to the article and approved the submitted version.

## Funding

This work was supported by the Vrije Universiteit Brussel (Methusalem grant), UZ Brussel (Scientific Fund Willy Gepts), the Research Foundation – Flanders (FWO)-Krediet aan Navorser (57090), and the NHMRC of Australia (1181516). Y.B. is a postdoctoral fellow of the FWO (62930).

## Conflict of Interest

The authors declare that the research was conducted in the absence of any commercial or financial relationships that could be construed as a potential conflict of interest.

## Publisher’s Note

All claims expressed in this article are solely those of the authors and do not necessarily represent those of their affiliated organizations, or those of the publisher, the editors and the reviewers. Any product that may be evaluated in this article, or claim that may be made by its manufacturer, is not guaranteed or endorsed by the publisher.

## Supplementary Material

Supplementary Figure 1 Control immunofluorescent stainings of murine adult testicular tissue. **(A)** Immunofluorescence analysis of cell death (terminal deoxynucleotidyl transferase-mediated dUTP nick end labeling, TUNEL, **A**) and the somatic testicular cells **(B–E)** and extracellular matrix (ECM; **C**) of the SSC niche: Leydig cells (ß-HSD, **B**), laminin (LN, **C**), Sertoli cells (SOX9, **D**), blood-testis-barrier (ZO1, **E**), and peritubular myoid cells (ACTA2, **B–E**) are shown. **(D,E)** Inset corresponds to boxed area. Bar = 100 μm. **(F,G)** Immunofluorescent stainings of the germ cell sub-types using the constitutive germ cell marker DDX4 **(F,G)** and the post-meiotic germ cell markers CREM **(F)** and PNA **(F)** or the meiotic marker γH2AX **(G)** to identify spermatocytes (**F**, red arrowhead), round spermatids (**F**, red arrow), elongated spermatids (**F**, red triangle), leptotene spermatocytes (**G**, white triangle, panel 1), zygotene spermatocytes (**G**, white arrow, panel 2), and pachytene spermatocytes (**G**, white arrowhead, panel 3). Bar = 100 μm (white).

Supplementary Figure 2 Characterization germline stem (GS) cell and two-layer scaffold (2LS) cultures. **(A,B)** GS colonies on a MEF feeder exhibiting EGFP fluorescence **(B)**. **(C)** Flow cytometric analysis of EGFP expression in the GS cell culture before mixing with primary testicular cells. **(D)** The EGFP+ cells of the GS cell culture showed the distinct forward scatter/side scatter profile of GS cells. **(E)** Chimeric cell mixtures attached to Cellink-RGD macropores (dotted lines) during short-term culture. Bar = 100 μm. **(F)** DDX4+ and EGFP+ cells were absent at the end of the long-term culture in Cellink 2LS. Bar = 100 μm.
