# Supplementary figures and images for: Long-Term Maintenance and Meiotic Entry of Early Germ Cells in Murine Testicular Organoids Functionalized by 3D Printed Scaffolds and Air-Medium Interface Cultivation
Source: Front Physiol. 2021 Dec 24;12:757565. doi: 10.3389/fphys.2021.757565 (PMC8739976; doi:10.3389/fphys.2021.757565)

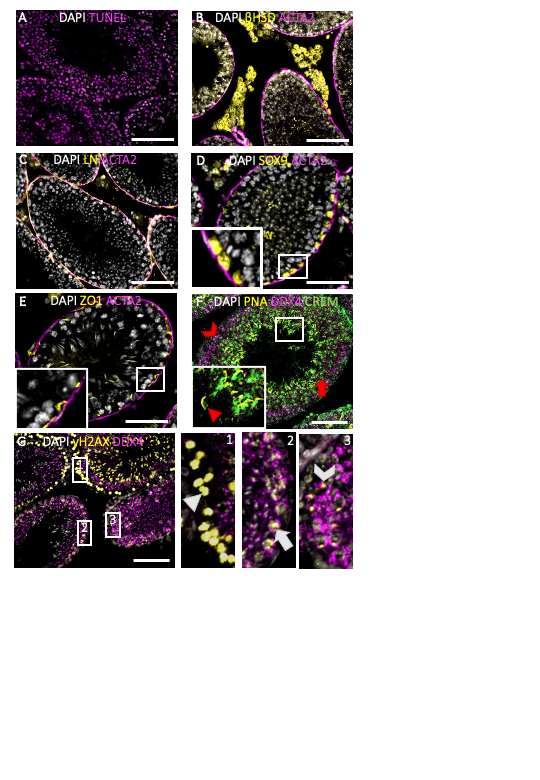

Supplement: Supplementary Figure 1 — Control immunofluorescent stainings of murine adult testicular tissue. (A) Immunofluorescence analysis of cell death (terminal deoxynucleotidyl transferase-mediated dUTP nick end labeling, TUNEL, A) and the somatic testicular cells (B–E) and extracellular matrix (ECM; C) of the SSC niche: Leydig cells (ß-HSD, B), laminin (LN, C), Sertoli cells (SOX9, D), blood-testis-barrier (ZO1, E), and peritubular myoid cells (ACTA2, B–E) are shown. (D,E) Inset corresponds to boxed area. Bar = 100 μm. (F,G) Immunofluorescent stainings of the germ cell sub-types using the constitutive germ cell marker DDX4 (F,G) and the post-meiotic germ cell markers CREM (F) and PNA (F) or the meiotic marker γH2AX (G) to identify spermatocytes (F, red arrowhead), round spermatids (F, red arrow), elongated spermatids (F, red triangle), leptotene spermatocytes (G, white triangle, panel 1), zygotene spermatocytes (G, white arrow, panel 2), and pachytene spermatocytes (G, white arrowhead, panel 3). Bar = 100 μm (white). [file Image_1.tiff]

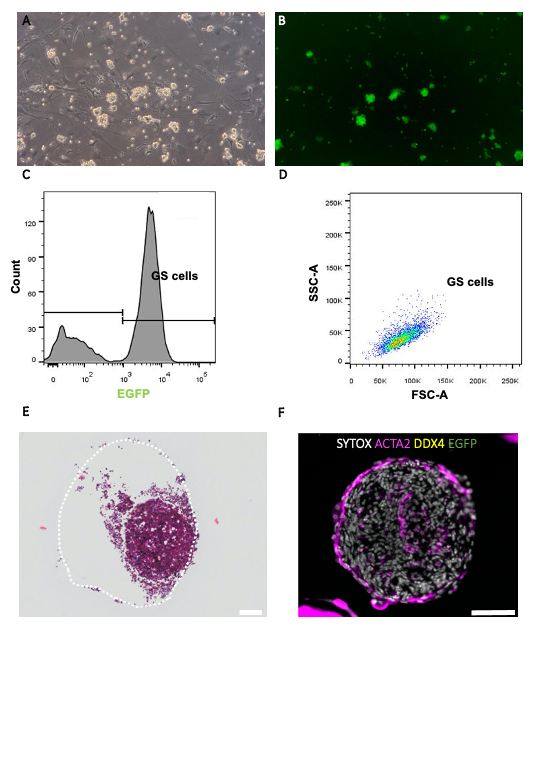

Supplement: Supplementary Figure 2 — Characterization germline stem (GS) cell and two-layer scaffold (2LS) cultures. (A,B) GS colonies on a MEF feeder exhibiting EGFP fluorescence (B). (C) Flow cytometric analysis of EGFP expression in the GS cell culture before mixing with primary testicular cells. (D) The EGFP+ cells of the GS cell culture showed the distinct forward scatter/side scatter profile of GS cells. (E) Chimeric cell mixtures attached to Cellink-RGD macropores (dotted lines) during short-term culture. Bar = 100 μm. (F) DDX4+ and EGFP+ cells were absent at the end of the long-term culture in Cellink 2LS. Bar = 100 μm. [file Image_2.tiff]
